# Factors Related to Locomotive Syndrome in School-Aged Children in Okazaki: A Cross-Sectional Study

**DOI:** 10.3390/healthcare9111595

**Published:** 2021-11-20

**Authors:** Yingzhi Gu, Tadashi Ito, Yuji Ito, Koji Noritake, Nobuhiko Ochi, Naomichi Matsunaga, Daiki Takahashi, Hideshi Sugiura

**Affiliations:** 1 Department of Integrated Health Sciences, Graduate School of Medicine, Nagoya University, Nagoya 461-8673, Japan; gu.yingzhi@a.mbox.nagoya-u.ac.jp (Y.G.); sanjigen@mikawa-aoitori.jp (T.I.); matsunaga.naomichi@icloud.com (N.M.); takahashi.daiki@g.mbox.nagoya-u.ac.jp (D.T.); 2 Three-Dimensional Motion Analysis Room, Aichi Prefectural Mikawa Aoitori Medical and Rehabilitation Center for Developmental Disabilities, Okazaki 444-0002, Japan; 3 Department of Pediatrics, Aichi Prefectural Mikawa Aoitori Medical and Rehabilitation Center for Developmental Disabilities, Okazaki 444-0002, Japan; yuji.ito@med.nagoya-u.ac.jp (Y.I.); aoi2pochi@yahoo.co.jp (N.O.); 4 Department of Orthopedic Surgery, Aichi Prefectural Mikawa Aoitori Medical and Rehabilitation Center for Developmental Disabilities, Okazaki 444-0002, Japan; noritake@mikawa-aoitori.jp

**Keywords:** child health, lifestyle, locomotive syndrome, musculoskeletal abnormalities, sedentary behavior

## Abstract

The relationship of locomotive syndrome with other physical characteristics and lifestyle habits in children has not been fully elucidated. The aim of this study was to assess the prevalence of children’s locomotive syndrome, and to determine its relationship with the above-mentioned factors. This was a cross-sectional study of 285 elementary school children who volunteered to participate in a medical checkup for physical function. Data was collected via medical examination, clinical measurements, and questionnaires. A multivariable logistic regression model was used to determine the relationship (odds ratios; ORs) of participants’ characteristics, physical functions, and other outcomes determined by questionnaire on locomotive syndrome. The following factors were related to locomotive syndrome: older age (OR = 1.421, 95% confidence interval [CI] [1.039, 1.945]), male sex (OR = 4.011, 95% CI [2.189, 7.347]), and more time spent watching television per day (OR = 1.281, 95% CI [1.001, 1.640]). These results may assist in the encouragement of children to perform appropriate physical activities and avoid unhealthy lifestyle habits, reducing the occurrence of locomotive syndrome.

## 1. Introduction

The World Health Organization pointed out that musculoskeletal conditions are typically characterized by persistent pain and limitations in mobility, dexterity, and functional ability affecting muscles, bones, joints, and associated tissues of the locomotor system [1]. Locomotive syndrome is a condition in which motor function is reduced because of limitations imposed on the motor organs by, e.g., trauma and musculoskeletal conditions such as pain, joint stiffness, muscle weakness, and reduced balance function [2]. This condition has been receiving increasing priority in Japan. Although the prevalence of musculoskeletal conditions increases with age, children are also affected [1]. Recently, as locomotive syndrome is generally observed in adults and older individuals, hypomotility and hypermobility has been recognized as causes. In one study, the proportion of children with this syndrome was 41.6% [3]. Thus, it warrants attention in terms of children’s musculoskeletal health status. Previous studies have revealed that more than 8% of children worldwide had musculoskeletal conditions, and that the number of consultations increased with age [4,5]. When researchers examined school-aged children who were clinically diagnosed with musculoskeletal conditions after undergoing a primary, questionnaire-based physical checkup, 44.4% had not reported related problems in the questionnaire [5]. This indicates that children may not be aware that they have locomotive syndrome; by implication, their parents would also be ignorant, and the prevalence of this condition among children may be underestimated. However, musculoskeletal conditions have a substantial impact on children’s health and quality of life, and may lead to musculoskeletal disorders such as trauma, arthritis, and osteoporosis [6]. Such disorders account for 4.9% of all pediatric admissions as the sixth most common cause of hospitalization among children and adolescents [7]. Therefore, the accurate assessment of locomotive syndrome in school-aged children is crucial.

The most common bodily locations affected by locomotive syndrome in children and adolescents who visit the hospital are the feet, knees, and back [4]. Moreover, trunk-muscle weakness and imbalance may cause low back pain [8]. Additionally, there is evidence that motor ability is associated with balance [9]. Furthermore, gait is considered an acceptable screening tool for musculoskeletal conditions in school-aged children [10]. In addition, obese children more frequently experience such conditions than normal-weight children [11] and sedentary behaviors, such as watching television (TV) and playing video games, are more likely to lead to obesity than more active behaviors [12]. According to the above, individual factors such as physical characteristics (body-fat percentage, bone-mineral density, muscle strength, balance, and gait) and lifestyle habits (a high amount of unfavorable sedentary behavior and a lack of physical activity) may be considered risk factors for locomotive syndrome. However, the relationship of this condition with physical characteristics and lifestyle habits in school-aged children has not been fully elucidated.

The purpose of this study was to clarify the relationship between locomotive syndrome and physical characteristics and lifestyle habits in school-aged children to assist in preventing locomotive syndrome, which leads to musculoskeletal disorders. We hypothesized that decreased physical function and an unfavorable sedentary lifestyle are associated with locomotive syndrome in children.

## 2. Materials and Methods

### 2.1. Participants

We performed a prospective, cross-sectional study, “Okazaki child medical checkup for physical function,” from January 2018 to March 2020. The research staff went to the elementary schools in Okazaki city and communicated the details of the study with the school instructors. Children aged 6–12 years were invited to partake in the medical checkups and were asked for permission to publicize the obtained data; the children made the decision with their parents whether to participate. Recruitment was conducted per mail; of the 1997 invited children, 437 underwent a medical checkup. 

The exclusion criteria were as follows: (1) an inability to complete the physical-function tests; (2) orthopedic, neurological, ophthalmological, auditory, respiratory, or cardiovascular abnormalities that may have affected the results of physical-function tests; (3) previously diagnosed autism spectrum disorder or attention deficit hyperactivity disorder; and (4) a substandard score for the Raven’s Colored Progressive Matrices [13] and the Peabody Picture Vocabulary Test-Revised (Nihon Bunka Kagakusha Co., Ltd., Tokyo, Japan), which indicates intellectual disability. Participants who met one or more of the exclusion criteria were excluded from the study. Therefore, 152 children were excluded and 285 were enrolled in this study.

Written informed assent and informed consent were obtained from all participants and their parents, respectively, prior to participants’ inclusion in the study, and this research was approved by the Ethics Committee of the Aichi Prefectural Mikawa Aoitori Medical and Rehabilitation Center for Developmental Disabilities (IRB approval number: 29002).

### 2.2. Data Collection

The checkup consisted of the following: a medical examination conducted by an orthopedic surgeon and a pediatric neurologist; clinical measurements made by trained pediatric physiotherapists; and questionnaires about lifestyle habits completed by the children and/or their parents.

#### 2.2.1. Health-Checkup Assessment Sheet

**Figure 1:**
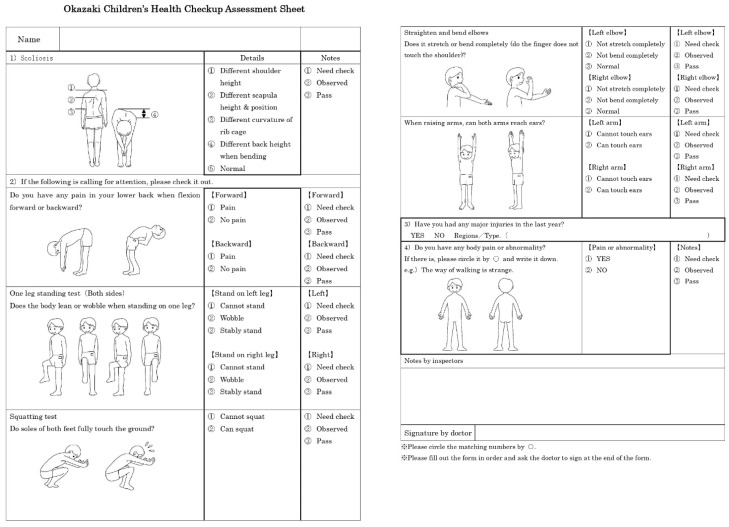
Okazaki Children’s Health Checkup Assessment Sheet. Participants were assessed for locomotive syndrome by using the musculoskeletal health checkup assessment sheet [4].

To assess participants’ locomotive syndrome, we used a musculoskeletal health-checkup assessment sheet (Figure 1) revised by Japan Clinical Orthopedic Association in 2016. The criteria assessed were as follows: (1) scoliosis, (2) forward and backward flexion, (3) standing on one leg, (4) squatting, (5) elbow-straightening and bending, (6) arm-raising, (7) injuries in the past year, and (8) current bodily pain or abnormalities. An abnormality in even one of these criteria indicated locomotive syndrome.

(1) Scoliosis

Children were requested to bend forward in the standing position until the upper body was horizontal. Any back or rib-cage abnormality visible in this position was considered a musculoskeletal condition [14]. This examination was completed by the physical therapist in our research team.

(2) Forward and backward flexion

Children were requested to bend forward and backward in the standing position. If they could not touch the ground with their fingertips when bending forward, or if bending backward as far as they could resulted in back pain, they were considered positive for a musculoskeletal condition [15].

(3) Standing on one leg

The one-leg standing time was measured on both sides in order to test children’s balance ability, and the longest time was selected for assessment. Children who could not stand motionless on one leg for at least 5 s were considered positive [15].

(4) Squatting

Children were requested to squat with their hands and arms stretched forward. If their heels could not fully touch the ground, or their center of gravity was unstable and resulted in an offset or falling, they were considered positive [5,15].

(5) Elbow-straightening and bending

Children were requested to straighten their arms in front of them while standing, with their palms turned up, and to bend the elbows in that position. If their arms could not be fully straightened, or if their hands could not touch their shoulders when bending at the elbow, they were considered positive [5,15].

(6) Arm-raising

If the children could not reach their ears when raising their arms in the standing position, they were considered positive [5,15]. This examination and that of elbow-straightening and bending were designed to test the range of motion of children’s upper limbs.

(7) Major injuries in the last year (yes/no)

An answer of “yes” was considered indicative of a musculoskeletal condition.

(8) Current body pain or abnormality (yes/no)

An answer of “yes” was considered indicative of a musculoskeletal condition.

#### 2.2.2. Measurement of Variables

(9) Body-fat percentage

A multi-frequency bioelectrical impedance analyzer (MC-780A, Tanita Corp., Tokyo, Japan) was used to measure body-fat percentage. The children were asked to stand on a platform containing electrodes anterior and posterior to the feet, while holding the hand electrodes in such a way that it was in contact with the palm and thumb of each hand. In this position, the arms extend naturally on both sides of the body. After several seconds, we recorded the children’s body fat percentage.

(10) Bone stiffness index

Bone-mineral density was examined by quantitative ultrasound using the A-1000 EXP II (GE Healthcare Japan Corp., Tokyo, Japan). The modes used included broadband ultrasound attenuation, which measures the frequency-dependent attenuation of ultrasound, and speed of sound, which reflects the transmission velocity of ultrasound through soft tissue [16]. One of the child’s feet was placed in the center of the machine, and the ankles and machine were moistened with alcohol. The bone stiffness index was derived from the bone-mineral density.

(11) Grip strength

Grip strength (in kg) was averaged for both hands using a Smedley spring-type handheld dynamometer (GRIP-D; Takei Scientific Instruments Co., Ltd., Niigata, Japan). The maximum grip strength was measured in the sitting position, with the elbow extended at 0°, the arm and torso aligned, and the wrist in a neutral position.

(12) Two-step value

In order to investigate children’s balance, they were instructed to take two steps, each as large as possible. The two-step value was determined as the ratio of this distance to their height [17].

(13) Gait deviation index (GDI)

The GDI is a common method of measuring gait pathology [18]. We measured it via instrumented, three-dimensional gait analysis with an eight-camera motion-analysis system (type MX-T 20S; Vicon Motion Systems Ltd., Oxford, UK), using a sampling frequency of 100 Hz. After the collection of anthropometric data, children were equipped with 16 retro-reflective markers (in combination with the Plug-In Gait lower body Ai model; Vicon) to obtain kinematics of the pelvis, hips, knees, and ankles on both sides. They were requested to walk on a walking mat while physiotherapists, experienced in clinical gait analysis, collected data [19]. The data from three trials for each child were exported from Polygon (Vicon Polygon 4.4; Vicon) to Microsoft Excel (Microsoft Corp., Redmond, WA, USA) [19], and averaged for subsequent data analysis.

(14) Gait speed

Children were requested to walk 10 m at a regular walking speed. This walking speed was recorded in m/s.

(15) Lifestyle-assessment questionnaire

Children and their parents responded to the following questions:

1. How much time does your child spend performing sports per week?
2. How much time does your child spend watching TV (including Internet content) per day?
3. How much time does your child spend playing video games per day?

### 2.3. Sample Size

The required sample size for performing a two-tailed Mann-Whitney U-test was determined using G*Power, with an alpha level of 0.05, a power of 0.95, and a large effect size (d = 0.5) [20,21]. Based on these assumptions, the required sample size was determined to be 220.

### 2.4. Statistical Analysis

The normal distribution for each variable was confirmed using the Shapiro–Wilk test. Participants’ data were compared using a two-tailed independent *t*-test or Mann–Whitney U-test. Fisher’s exact test (two-tailed) was used to compare differences in proportions between the sexes in each group (children with and those without locomotive syndrome). Logistic regression analysis was used to examine the association of age, sex, height, weight, body mass index (BMI), body-fat percentage, stiffness index, grip strength, two-step value, GDI, gait speed, time spent performing sports per week, time spent playing video games per day, and time spent watching TV per day, with locomotive syndrome. *p*-values < 0.05 were considered statistically significant. A multivariable logistic regression model was used to determine odds ratios for age, sex, height, body-fat percentage, stiffness index, grip strength, two-step value, GDI, gait speed, time spent performing sports per week, time spent playing video games per day, and time spent watching TV per day in terms of locomotive syndrome, controlling for confounding factors (age, sex, and height). All data management and statistical computations were performed using IBM SPSS Statistics for Windows 24.0 (IBM Corp., Armonk, NY, USA).

## 3. Results

**Figure 2:**
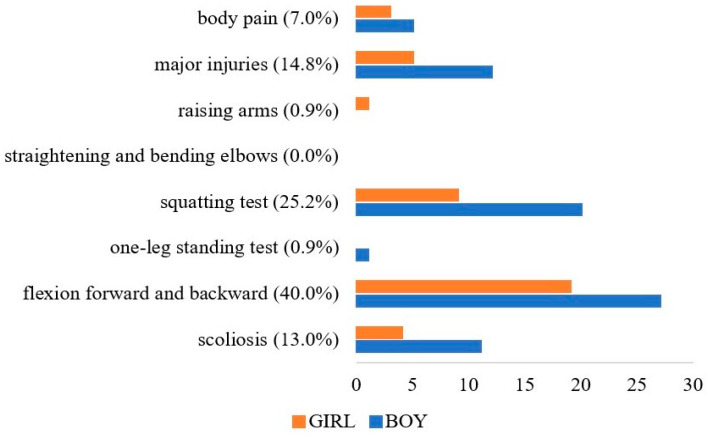
The proportion participants with each musculoskeletal condition. These data were obtained among children with locomotive syndrome by performing musculoskeletal examination.

The health checkup revealed that children with locomotive syndrome accounted for 40.4% (115/285) of those who were assessed. The most common musculoskeletal condition among the children examined was difficulty bending forward and backward, accounting for 40.0% (46/115) of the conditions (Figure 2). No children experienced problems with straightening or bending their elbows. Problems with arm-raising and standing on one leg were also very rare, accounting for 0.9% of all cases of locomotive syndrome. The only condition in which girls were affected to a higher degree was forward and backward flexion.

The characteristics of the participants, and comparisons between children with and those without locomotive syndrome, are summarized in Table 1. Characteristics in which there were significant differences between the two groups were age (*p* = 0.004), sex (*p* = 0.001), height (*p* = 0.002), weight (*p* = 0.001), and BMI (*p* = 0.002). The physical function and questionnaire responses of the participants, and comparisons between children with and those without locomotive syndrome, are summarized in Table 2. Variables in which there were significant differences between the two groups were time spent watching TV per day (*p* = 0.022), and time spent playing video games per day (*p* = 0.002).

In the multivariable analysis, the following variables were significantly related to locomotive syndrome: older age (odds ratio [OR] = 1.421, 95% confidence interval [CI] [1.039, 1.945]), male sex (OR = 4.011, 95% CI [2.189, 7.347]), higher body-fat percentage (OR = 1.064, 95% CI [1.014, 1.117]), weaker grip strength (OR = 0.854, 95% CI [0.757, 0.964]), and more time spent watching TV per day (OR = 1.281, 95% CI [1.001, 1.640]) (Table 3). Thus, locomotive syndrome is related to age, sex, body-fat percentage, grip strength, and time spent watching TV per day.

## 4. Discussion

In this cross-sectional study, we demonstrated which physical characteristics and lifestyle habits were associated with locomotive syndrome in school-aged children. Children with locomotive syndrome accounted for slightly less than half of the participants, and the majority were boys and in higher grades. Those children also had higher BMI values, as well as more hours spent watching TV than children without locomotive syndrome. Locomotive syndrome was statistically significantly related to age, sex, body-fat percentage, grip strength, and time spent watching TV and playing video games. These results support our hypothesis and indicate that it is important for school-aged children to maintain an active healthy lifestyle to prevent locomotive syndrome.

Body-fat percentage was associated with locomotive syndrome. Children with this condition also had a higher BMI than those without, although these were not associated upon multivariable analysis. Although dieting is not recommended for weight loss in children who are growing and developing, weight control by increasing physical activities and improving eating habits is important for overweight children. However, there was no significant relationship between time spent performing sports per week and the prevalence of locomotive syndrome in this study. According to the existing research, the obesity rate of rural children, who eat less fruit and more dairy products than urban children, is higher, even if they exercise more than urban children do [22]. Given that obese children are more likely to develop locomotive syndrome, unhealthy dietary habits and imbalanced nutrition may contribute to this condition [11].

Children with locomotive syndrome spent more hours watching TV, and time spent watching TV and playing video games was related to this condition. In modern society, the abundance of TV programs and Internet content increases children’s access to information and entertainment; at the same time, children with a lack of self-discipline can easily become addicted to such content. Related studies have revealed that frequent web-browsing and TV/movie-watching may increase the risk of musculoskeletal discomfort [23,24,25]. According to the result of multivariable analysis in this study, no correlation was showed among the time of playing video games, which is generally considered to be a sedentary behavior as well as watching TV. This could be because children are in a more excited state when playing video games. Coupled with the emergence of video games that require physical activity, video games may need to be fade away from the category of sedentary behaviors. It was reported that caregivers should learn to recognize signs of musculoskeletal disorders in children and promptly seek appropriate care [26]. Reducing the amount of time that a child spends watching TV and playing video games may also help reduce such conditions.

It is worth noting that the time spent watching TV is associated with the risk of overweight and obesity in children [12]. As both these variables were included in our multivariable model, the relationship between body fat percentage and time spent watching TV needs to be considered in future studies.

Grip strength did not differ statistically significantly between the two groups in this study; however, grip strength was related to locomotive syndrome. This correlation may also reflect a relationship between overall muscle strength and locomotive syndrome among the studied children. Locomotive syndrome may weaken a child’s grip strength and even their whole-body muscle strength. In order to reduce the occurrence of such weakness, therefore, children should perform appropriate physical activities to increase their muscle strength, regardless of whether they have locomotor syndrome.

Children with locomotive syndrome in this study were mostly boys and in higher grades; both sex and age were associated with this condition. In previous studies, it was demonstrated that boys performed more moderate-to-vigorous physical activities than girls, and that they were at higher risk of injury [27,28]. As for age, primary-school students typically take part in more sports-club activities as they increase in grade, which probably increases the possibility of injuries. Furthermore, an increase in schoolwork with age can contribute to prolonged periods of sitting by senior students [27], which is consistent with the findings of this study in terms of the relationship between an unfavorable sedentary lifestyle and the prevalence of locomotive syndrome. In contrast with the situation in children, although the incidence of locomotive syndrome also increased with age in a study of adults, including older individuals, the condition was more common among women [29]. This may be because of the speed of decline in bodily function.

Nearly half of the participants in this study had locomotive syndrome; impaired forward and backward flexion accounted for approximately three-quarters of these cases, almost all in forward flexion. This is consistent with the results of a previous study, where forward flexion was the most common this condition in elementary-school students [15]. Impairment in forward flexion may be related to the length and stretching of the hamstring muscles [30]. However, as the items used to determine locomotive syndrome in children were not fully validated, unlike those for adults, a future longitudinal study is needed to confirm this hypothesis.

The present study has several limitations. First, we recruited participants from Okazaki City only, and it would be beneficial to investigate a wider area in order to improve the generalizability of the findings. Second, we did not assess the relationship between locomotive syndrome and eating habits, which are also lifestyle habits. Moreover, the questionnaire used in this study was subjective, with the possibility that the participants had overoptimistic views of their lifestyles. Third, as this was an observational study, the causality of the associations between locomotive syndrome and individual characteristics or lifestyle habits could not be determined: therefore, there may be residual confounding effects in the risk estimates. Finally, as this was a cross-sectional study, no inferences about causality can be made from our results. From the literature, it can be observed that repeated injuries in children are common [31]. Hence, it is difficult to say whether locomotive syndrome and musculoskeletal problems in children are contributing to increased sedentary time and decreased physical activity, resulting in obesity, decreased muscle strength, and other problems. A longitudinal study in which e.g., the control variate method is used is needed to elucidate sequential changes in locomotive syndrome and their association with physical characteristics or lifestyle habits.

## 5. Conclusions

In conclusion, we discovered that locomotive syndrome in school-aged children was associated with male sex, increasing age, higher body-fat percentage, lower grip strength, and a longer time spent watching TV/Internet content. These results indicated that it is important to assess children’s body fat percentage, grip strength, and time spent watching TV to prevent the development of locomotive syndrome in this population.

## Figures and Tables

**Table 1:** Characteristics of participants.

Variables	Children with Locomotive Syndrome (*n* = 115)	Children without Locomotive Syndrome (*n* = 170)	*p*-Value
Age (years) ^1^	9 (6–12)	8 (6–12)	0.004 *
Sex, male/female ^2^	76/39	68/102	0.001 *
Height (cm) ^1^	133.0 (106.5–164.2)	127.8 (108.8–158.2)	0.002 *
Weight (kg) ^1^	28.7 (16.1–57.7)	25.1 (16.6–59.6)	0.001 *
Body mass index (kg/m^2^) ^1^	16.0 (13.1–26.6)	15.2 (13.0–24.2)	0.002 *

^1^ Analysis performed with Mann–Whitney U-test. ^2^ Analysis performed with chi-square test. * Significant at *p* < 0.05. Data are presented as median (range), except for sex.

**Table 2:** Physical function outcomes and questionnaire.

Variables	Children with Locomotive Syndrome (*n* = 115)	Children without Locomotive Syndrome (*n* = 170)	*p*-Value
Body fat percentage (%)	12.9 (3.9–42.7)	11.7 (3.3–35.7)	0.159
Stiffness index	81.7 ± 11.4	80.3 ± 12.6	0.205
Grip strength (kg)	12.0 (5.25–21.4)	11.0 (5.6–26.8)	0.142
Two-step value	1.6 (0.8–2.0)	1.6 (1.0–1.9)	0.386
Gait deviation index	94.1 ± 7.4	94.5 ± 7.1	0.586
Gait speed (m/s)	1.2 (0.7–1.8)	1.1 (0.8–1.7)	0.405
Physical activity (h/week)	5.0 (0–20.0)	4.0 (0–25.5)	0.127
Watching television (h/day)	2.0 (0.5–10.0)	1.5 (0–9.0)	0.022 *
Playing video games (h/day)	0.5 (0–4.0)	0.5 (0–7.0)	0.002 *

Analysis performed with the Mann–Whitney U-test or the independent *t*-test. * Statistically significant at *p* < 0.05. Data are presented as median (range) or mean ± standard deviation.

**Table 3:** Multivariable analysis for factors associated with locomotive syndrome among the participants (*n* = 285).

Variables	Odds Ratio	95% Confidence Interval	*p*-Value ^1^
Age	1.421	1.039–1.945	0.028 *
Sex (male)	4.011	2.189–7.347	0.001 *
Height	1.040	0.986–1.098	0.150
Body fat percentage	1.064	1.014–1.117	0.012 *
Stiffness index value	0.985	0.959–1.011	0.256
Grip strength	0.854	0.757–0.964	0.010 *
Two-step value	0.993	0.978–1.008	0.358
Gait deviation index	0.996	0.960–1.033	0.836
Gait speed	0.589	0.122–2.838	0.510
Physical activity	1.012	0.949–1.080	0.715
Watching television	1.281	1.001–1.640	0.049 *
Playing video games	0.982	0.663–1.457	0.929

The variables in the table were included in the multivariable model as explanatory variables. ^1^ Result of logistic regression analysis. * Statistically significant at *p* < 0.05.

## Data Availability

All relevant data are presented within the manuscript. All data are available from the corresponding author on reasonable request.
